# NOX2 and NOX4 expression in monocytes and macrophages-extracellular vesicles in signalling and therapeutics

**DOI:** 10.3389/fcell.2024.1342227

**Published:** 2024-04-16

**Authors:** Deepak Rathi, Claudio Rossi, Pavel Pospíšil, Renuka Ramalingam Manoharan, Luigi Talarico, Agnese Magnani, Ankush Prasad

**Affiliations:** ^1^ Department of Biophysics, Faculty of Science, Palacký University, Olomouc, Czechia; ^2^ Department of Biotechnology, Chemistry and Pharmacy, University of Siena, Siena, Italy; ^3^ Center for Colloid and Surface Science (CSGI), Florence, Italy

**Keywords:** extracellular vesicles, NADPH oxidase, NOX2, NOX4, monocytes, macrophages, reactive oxygen species, free radicals

## Abstract

Extracellular vesicles (EVs) are a type of cytoplasmic vesicles secreted by a variety of cells. EVs originating from cells have been known to participate in cell communication, antigen presentation, immune cell activation, tolerance induction, etc. These EVs can also carry the active form of Nicotinamide Adenine Dinucleotide Phosphate Oxidase Hydrogen (NADPH) oxidase, which is very essential for the production of reactive oxygen species (ROS) and that can then modulate processes such as cell regeneration. The aim of this study is to characterize the EVs isolated from U-937 and THP-1 cells, identify the NADPH oxidase (NOX) isoforms, and to determine whether EVs can modulate NOX4 and NOX2 in monocytes and macrophages. In our study, isolated EVs of U-937 were characterized using dynamic light scattering (DLS) spectroscopy and immunoblotting. The results showed that the exogenous addition of differentiation agents (either phorbol 12-myristate 13-acetate (PMA) or ascorbic acid) or the supplementation of EVs used in the study did not cause any stress leading to alterations in cell proliferation and viability. In cells co-cultured with EVs for 72 h, strong suppression of NOX4 and NOX2 is evident when monocytes transform into macrophagic cells. We also observed lower levels of oxidative stress measured using immunoblotting and electron paramagnetic resonance spectroscopy under the EVs co-cultured condition, which also indicates that EVs might contribute significantly by acting as an antioxidant source, which agrees with previous studies that hypothesized the role of EVs in therapeutics. Therefore, our results provide evidence for NOX regulation by EVs in addition to its role as an antioxidant cargo.

## 1 Introduction

Cells (prokaryotes or eukaryotes) secrete lipid-bound vesicles that encapsulate nucleic acids and proteins into the extracellular space called EVs (Di Bella, 2022). The size ranges for these EVs are known to range from nanometres to micrometres. EVs are a broad term used to describe a heterogeneous group of vesicles that are released into the extracellular space by cells; these can be exosomes, microvesicles (also called exosomes or shedding vesicles), and apoptotic bodies (Johnson et al., 2020). Cells can secrete different types of vesicles that can be heterogenic in size and composition (Surman et al., 2017). The biogenesis pathways of these vesicles can vary. EVs can be isolated from different body fluids such as saliva, urine, plasma, lymph, synovial fluid, cerebrospinal fluid, amniotic fluid, breastmilk, tears, bile, gastric acid, etc. (Doyle and Wang, 2019). During the last two decades, research related to EVs has accelerated due to its supposed role in different physiological pathways, cell communication, drug delivery, and as a therapeutic compound (Nederveen et al., 2021).

Biogenesis of EVs can be broadly classified into microvesicles or exosome biogenesis. Microvesicles, also known as exosomes or shredding vesicles, are formed by outward budding of the plasma membrane of the cells. The formation of microvesicles can be triggered by stressors or any change in the microenvironment. The change in lipid composition and the action of the cytoskeleton component results in outward budding and eventually scission through the formation of the neck-like structure. During the budding process, different components of the cell such as proteins, lipids, and nucleic acids can be loaded, referred to as cargo. The process of exosome biogenesis begins with endocytosis, in which extracellular components or materials internalize into the cells. Following this step, the structure is directed toward the lysosomes or the cell surface (van Niel et al., 2018; Kalluri and LeBleu, 2020; Lau and Yam, 2023). The biogenesis process is significant for the characterization of exosomes because some proteins are incorporated in exosomes, which include some tetraspanin, lipid bilayer tetraspanin proteins such as CD9, CD63 and CD81 along with the intraexosomal proteins tumour susceptibility gene 101 (TSG101) intraexosomal proteins, the ALG2 interacting protein X (ALIX); however, to date, no single specific marker that has been discovered that can define only exosomes and differentiate them from microvesicles (Barile and Vassalli, 2017). The biogenesis and secretion of intraluminal vesicles are determined by a multiunit cytoplasmic system called the endosomal sorting complex required for transport (ESCRT), which modulates the plasma membrane for vesicle budding and microvesicular cargo sorting.

In recent research, it has also been evident that exosomes produced by tumor cells can modulate the cancer’s progression, and exosomes have also been shown to facilitate the metastasis of cancer to secondary organs (Chen et al., 2021). In lung cancer models, cancer cell exosomes can suppress the effect of innate immunity, resulting in suppression of immunity against virus infection (Gao et al., 2018). In this process of suppression of immunity against viral infection, the epidermal growth factor receptor (EGFR) carrying exosomes releases to host macrophages in which these EGFR carrying exosomes suppress the expression of type 1 interferon and interferon regulatory transcription factor 3 (IRF3) expression. In human fibroblasts, exosomes play a crucial role in removing harmful cytosolic DNA to maintain cellular homeostasis. However, artificial inhibition of exosome secretion can lead to the accumulation of genomic DNA in the cytoplasm. Subsequently, DNA sensing proteins may activate the innate immune response, resulting in ROS-dependent DNA damage, which in turn can lead to cell cycle arrest or apoptosis (Takahashi et al., 2017).

NADPH oxidases are one of many sources of reactive oxygen species (ROS) in biological systems. There are seven isoforms (Nox1–5, Duox1, Duox2) that have different tissue distribution within the cell or organelles. (Bedard and Krause, 2007; Mittler et al., 2011; Landry and Cotter, 2014; Krishnamoorthy and Chang, 2018; Hahner et al., 2020; Prasad et al., 2021). This ROS generation, in addition to being involved in the elimination of the invading pathogen, is also known to regulate several signaling pathways. Under conditions where ROS, such as the superoxide anion radical (O_2_^•-^) and hydroxyl radical (HO^•^), are formed above a certain threshold that cannot be scavenged by enzymatic and non-enzymatic antioxidants within the cells, peroxidation of polyunsaturated fatty acids (PUFA’s) can occur. This can then lead to the generation of reactive intermediates and products such as malondialdehyde (MDA) (Ayala et al., 2014; Tsikas, 2017; Pospisil et al., 2019; Dalrymple et al., 2022). Generated MDA is a well-known marker of oxidative stress and lipid peroxidation; however, it can also be involved in the post-translational modification of proteins, through the addition of carbonyl groups (-CO) which is known as carbon carbonylation (Suzuki et al., 2010; Tola et al., 2021). Understanding the regulation and function of NADPH oxidase is an active area of research because of its potential therapeutic implications. In recent results presented by Hervera et al., it has been shown that NOX2 present in macrophage secreted exosomes was involved in the regulation of axonal regeneration of injured axons; therefore, NOX regulation may serve as potential factor in regenerative medicine (Hervera et al., 2018).

Our study aims to characterize EVs isolated from U-937 and THP-1 cells, identification of NOX isoforms, and whether EVs can modulate NOX4 and NOX2 expression in monocytes and macrophages. The motivation behind the study is based on the fact that NOX4 expression has been reported predominantly in monocytes, but it has been known to be present in M2 macrophages (post-inflammatory); the same is true for part of the population of M1 macrophages (pre-inflammatory) that express the NOX2 complex (Bermudez et al., 2016). M1 and M2 macrophages are two distinct phenotypes of macrophages, whose functional profiles represent different activation states and immune system functions. M1 can be activated by bacterial lipopolysaccharide and interferon γ. After activation, M1 macrophages produce ROS and nitric oxide (NO) that helps in innate immunity, whereas M2 macrophages are activated through certain cytokines that induce collagen production for wound healing or tissue repair. In a recent study, miRNAs have been hypothesized to inhibit the expression of divalent metal transporter 1 in cardiomyocytes, thus increasing glutathione (GSH) levels and depleting ROS and MDA formation (Zhang et al., 2022). Therefore, in addition to NOX expression, we also focused on protein modification and ROS suppression in monocytes and macrophages by co-culture experiments in the absence and presence of EVs.

## 2 Materials and methods

### 2.1 Reagents and antibodies

Cell culture medium (RPMI-1640), fetal bovine serum, and antibiotics [antibiotic-antimycotic solution] were purchased from Biosera (Nuaille, France). Phorbol 12-myristate 13-acetate (PMA), ascorbic acid, and polyethylene glycol 6,000 and 4-pyridyl-1-oxide-N-tert-butylnitrone (POBN) were obtained from Sigma Aldrich (St. Louis, Missouri, United States of America). Rabbit polyclonal anti-malondialdehyde (MDA) antibody and anti-NADPH oxidase 4 (anti-NOX4), were purchased from Abcam (Cambridge, United Kingdom) and HRP-conjugated goat anti-rabbit antibody was purchased from BioRad. CD63 monoclonal antibodies, NOX2 polyclonal antibody, and secondary antibody (HRP-conjugated goat anti-mouse) were obtained from Proteintech (GmbH Germany). Protease and phosphatase inhibitors were purchased from Roche (Mannheim, Germany) and Protein A-Agarose (sc-2001) was purchased from Santa Cruz Biotechnology (Heidelberg, Germany). Please refer to the Supplementary Material for a catalog of reagents and clones of antibodies.

### 2.2 Cell line and growing condition

**FIGURE 1:**
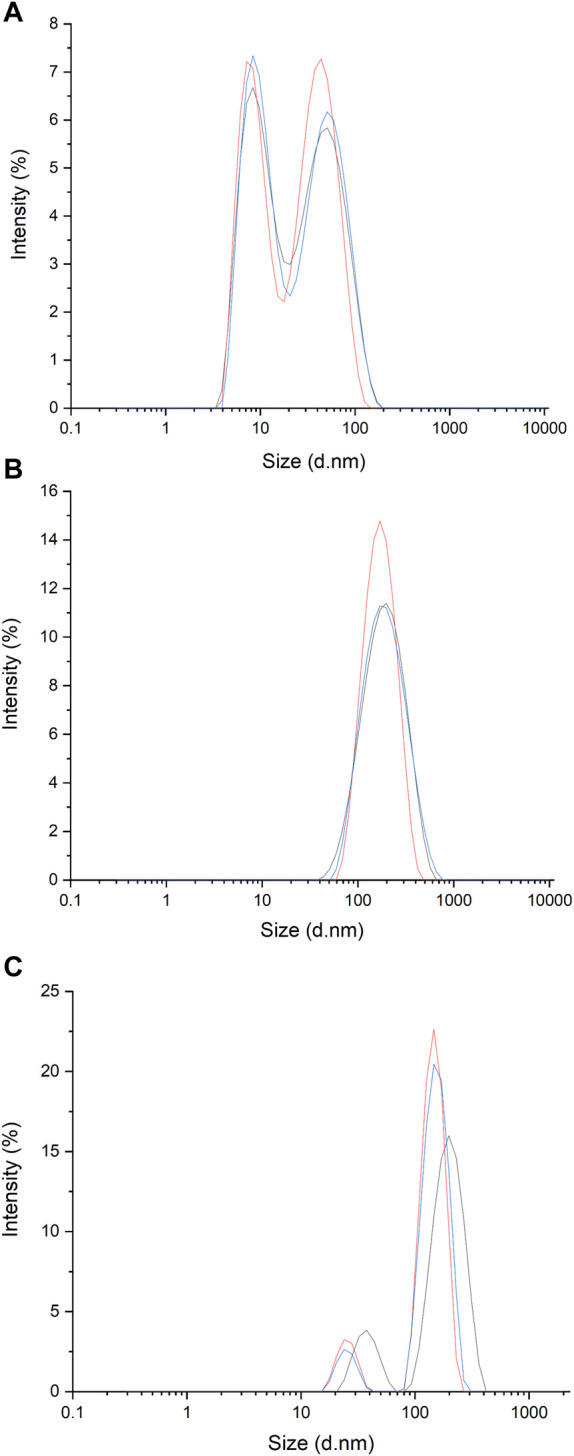
Spectra of dynamic light scattering (DLS) measurements of EVs present in filtered media (FBS was filtered through 0.2 μm filter) **(A)**; EVs isolated from media obtained from U-937 cell culture following filtration and centrifugation **(B)** and EVs present in media obtained from U-937 cell culture and following filtration and PEG enrichment **(C)**.

U-937 and THP-1 cells were purchased from the American Type Culture Collection (ATCC; Rockville, Maryland, United States) and cultured in RPMI 1640 medium containing 1% antibiotic v/v and 10% fetal bovine serum (FBS). To avoid contamination of EVs present in FBS, EV-depleted FBS was used. Using a 0.2 μm polyether sulfone membrane syringe filter (VWR International, Puerto Rico, United States of America), particles larger than 0.2 μm were removed (Shelke et al., 2014), and the filtrate was collected and measured (Shu et al., 2021) (Figure 1). Furthermore, to get rid of the smaller component <0.2 μm (Figure 1A), centrifugation was performed at 21,000 × *g* for 3 h and the supernatant was collected. Following this step, the obtained FBS was used for further cell culture.

### 2.3 Isolation of EVs from the media of cultured cells using centrifugation

From the media containing cultured cells (U-937) in the log phase (5 days after cell passage) and with viability ∼90–95%, the cell products were harvested and subjected to centrifugation at 300 *g* for 10 min at 4°C for removal of cells from the harvested media. The supernatant was collected and filtered through a 0.2 µm polyether sulfone membrane syringe filter and then centrifuged at 21,000 × *g* for 3 h at 4°C. After this, the supernatants were discarded, and the pellet was washed again at 21,000 × *g* (2X, 3 h) with ice-cold phosphate buffer saline (PBS, 1X). Subsequently, the pellet was suspended in 250 µL of HPLC grade water. The isolated EVs were stored at -20°C until further processing. The same procedure was followed for THP-1 cells.

### 2.4 Isolation of EVs by polyethylene glycol (PEG) precipitation

The medium containing cultured cells and cell products was harvested and then centrifugation was carried out at 300 × *g* for 10 min at 4°C for cell removal from the harvested medium. The supernatant was collected and filtered through 0.2 µm polyether sulfone membrane syringe filter. 50% of the PEG6000 solution was prepared in d/w, after which the filtrate was mixed with the PEG: filtrate (1: 2.5) followed by a short vortex and incubated at 4°C overnight to form aggregates. After overnight incubation, the PEG mixture was centrifuged at 13,000 *g* for 60 min at 4°C. The pellet was then washed twice with ice-cold PBS at 13,000×*g*. The collected pellet was stored at -20°C until further processing.

### 2.5 Characterization of EVs using dynamic light scattering (DLS) spectroscopy

To determine the size distribution of isolated particles in the suspension, DLS measurements were performed using a Malvern Zetasizer RED Pro using a detector angle of 173° (backscatter) at a temperature of 25°C. For sampling, 25 μL of isolated vesicles were diluted in 975 mL of d/w and particle size analysis was carried out for both EVs isolated using centrifugation (as in Section 2.3) and PEG enrichment (as in Section 2.4). Control measurements were performed using EV -free FBS. To confirm that our isolate contains an EV, we used a transmission electron microscope (TEM) to visualize the particles (data not shown).

### 2.6 Protein isolation from EVs

Based on the results obtained from the basic characterization of the isolated EVs, since potential aggregation was observed in EVs prepared using PEG enrichment (Figure 1C), further studies were carried out using EVs isolated using centrifugation. For protein isolation, EVs (in d/w) were sonicated (40% amplitude; 1 cycle for 30 s) in ice-cold Radioimmunoprecipitation Assay (RIPA) buffer [150 mM NaCl, 50 mM Tris (pH 8.0), 0.5% sodium deoxycholate, 0.1% SDS, 1% NP-40] containing 1% protease inhibitor and phosphatase inhibitor. Sonication was repeated 6 times with a 30 s gap before the next sonication. EVs were kept on ice after each cycle of sonication. Following this step, centrifugation at 18,400 × *g* was done for 30 min at 4°C to remove cell debris and to obtain proteins in the supernatant.

Estimation of protein was done using Pierce bicinchoninic (BCA) protein estimation kit (Thermo Fisher Scientific, Paisley, United Kingdom). The measurement procedure used is as outlined in the manufacturer’s guidelines with minor modifications as described in our previous study (Manoharan et al., 2023; Prasad et al., 2023).

### 2.7 Anti-CD63 blotting (surface marker) for EVs confirmation

Immunoblotting was performed with CD63 surface markers on EVs. The samples were prepared with 5 × Laemmli sample buffer along with 100 mM Dithiothreitol (DTT) and a protein concentration of 10 μg was used for electrophoresis. Prior to loading the samples, the protein samples were incubated at 70°C for 10 min in a dry bath. Subsequently, the sample was loaded onto 10% Tricine SDS-PAGE. The proteins were then transferred to either PVDF membrane (Bio-Rad, California, United States of America) which was charged prior with methanol or nitrocellulose membrane. The protein transfer to the membrane was achieved using the Trans-Blot Turbo transfer system (Bio-Rad, California, United States). Following the transfer, to ensure that the loading of protein samples is uniform (loading control), Ponceau staining (0.1% Ponceau S: 1% acetic acid, *w: v;* diluted to 100 mL d/w) was done. After washing, the membrane was blocked with 5% BSA in Tris-buffered saline (TBS) (pH 7.4) and 0.1% Tween 20 (referred to as TBST) for 90 min at room temperature (RT). After blocking, washing with TBST (3x) was done for 10 min each. The blocked membranes were then incubated for 90 min at RT with anti-CD63 mouse monoclonal antibody (dilution 1: 5000) followed by 3x washing (10 min each) with TBST. Subsequently, the membrane was incubated for another 90 min at RT with HRP-conjugated goat anti-mouse secondary antibody (dilution 1:10,000) and washed with TBST (3x, 10 min each). Immunocomplexes were imaged using the Amersham imager 600 (GE Healthcare, United Kingdom) and Immobilon Western Chemiluminescent HRP Substrate (Sigma Aldrich, GmbH, Germany).

### 2.8 Co-culture of U-937 cells with differentiating agents and EVs

Co-culture experimental setups were made. For the setting of the plates, U-937 and THP-1 controls with differentiating agents [250 nM or 150 nM PMA and ascorbic acid (5 μΜ and 10 μΜ)] were prepared. For the experimental sample, cells (1×10^5^/mL) under the above conditions (PMA or ascorbic acid) were cultured in the presence of exogenous EVs (20 μL, the same isolates were used for all experiments within the setup). Cells were treated with PMA/ascorbic acid for 72 h (for monocyte to macrophage differentiation) in a 6 well plate and isolated EVs (section 2.3) were added at the start of the treatment. Cell viability at 24h and 72 h were monitored using the trypan blue test to follow cell proliferation and effect of EVs and/or differentiating agents. For protein isolation, 5 mL of non-treated and treated cells were used, and quantification was done based on the method described in Section 2.6. Following quantification, 10 μg protein sample/lane was used for immunoblotting.

### 2.9 Western blot analysis

Immunoblotting was performed against NOX4 and NOX2. Until the blocking of membranes, the procedure as described in Section 2.8 was followed. The blocked membranes were then incubated for 90 min at room temperature (RT) with either anti-NOX4 rabbit monoclonal antibody (dilution 1:5000) or anti-NOX2 mice polyclonal antibody followed by 3x washing (10 min each) with TBST. Subsequently, the membranes were incubated for another 90 min at RT with HRP-conjugated goat anti-rabbit secondary antibody and HRP-conjugated goat anti-mice secondary antibody, respectively (dilution 1:10,000) and washed with TBST (3x, 10 min each). Immunocomplexes were imaged using the procedures described in Section 2.7.

To confirm MDA-protein adduct formation, nitrocellulose membranes transferred with MDA adduct proteins were incubated with rabbit polyclonal anti-malondialdehyde antibody prepared at a dilution of 1:5000 in TBST and incubated for 90 min at RT. Following three washes with TBST, the membranes were incubated with horseradish peroxidase (HRP) conjugated anti-rabbit secondary antibody (1:10,000) for 90 min at RT. The blots were imaged as described above.

### 2.10 EPR spin-trapping spectroscopy

Chemically generated HO^•^ using Fenton reagent was detected by spin-trapping with 10 mM POBN (4-pyridyl-1-oxide-N-tert-butylnitrone) containing 170 mM ethanol. The measurements were performed using an electron paramagnetic resonance spectrometer (MiniScope MS400, Magnettech GmbH, Berlin, Germany) (Pou et al., 1994). Experiments were carried out in the absence and presence of different concentrations of EVs isolated from U-937 cells resuspended in HPLC grade water. EPR spectra were recorded under the following parameters: microwave power (10 mW), modulation amplitude (1 G), modulation frequency (100 kHz), sweep width (100 G), scan rate (1.62 G s^–1^).

## 3 Results and discussion

### 3.1 Characterization of EVs using DLS

Using the dynamic light scattering method, we measured the mean Z-average (mean hydrodynamic size) of the sample obtained by centrifugation from U-937 cells. For the control (filtered and centrifuged FBS), the Z-average was recorded to be 15.7 nm. More than one peak with a polydispersity index of 0.43 was observed (Figure 1A; Table 1). It can be seen that in the sample isolated by centrifugation based on the method described by Shelke and co-workers (with minor modifications) (Shelke et al., 2014), a Z-average of ∼154.8 nm was observed with a standard deviation of about 15.2 nm and a polydispersity index of 0.30 (Figure 1B; Table 1). The presence of multiple peaks in the case of PEG samples can be attributed to the centrifugation, aggregation, and orientation of the vesicle (Figure 1C) (Genneback et al., 2013). As the sample acquired by the centrifugation method includes EVs falling within the 30–200 nm range and exhibits the presence of the CD63 marker, it is a likelihood that the isolates may be classified as exosome particles. However, we are uncertain due to a lack of understanding about their origin.

**TABLE 1:** The table shows the size distribution of particles within the samples measured using DLS.

Sample name	Size (nm)	Pdi
Filtered and centrifuged FBS	15.7 ± 0.3	0.43 ± 0.01
Exosomes (centrifuged)	154.8 ± 15.2	0.308 ± 0.04

### 3.2 Identification of extracellular markers specific for vesicles

**FIGURE 2:**
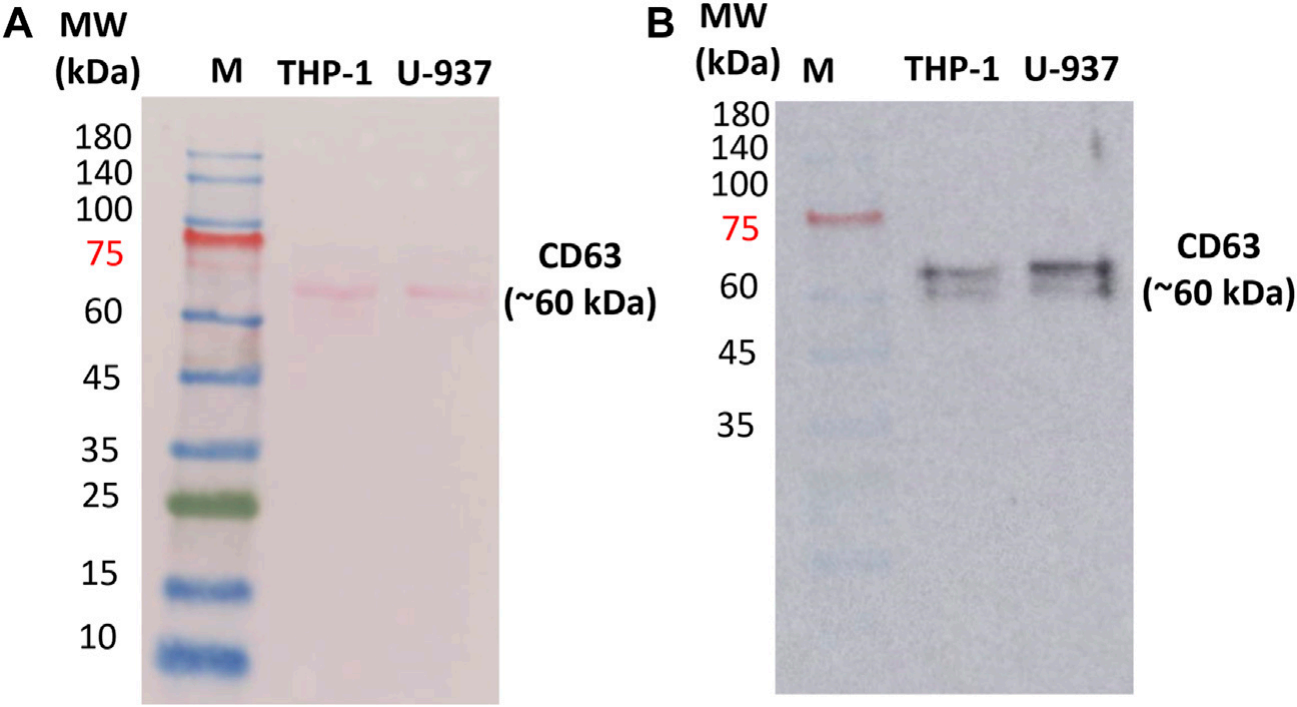
Ponceau staining of the nitrocellulose membrane to confirm the presence of proteins **(A)** and Western blot confirming the expression of transmembrane proteins CD63 in isolated EVs **(B)**. 10 µg of protein isolated from exosomes (U-937 and THP-1) were loaded. ‘M’ indicates the prestained marker of which 5 μL was loaded. CD63 primary antibody (1: 2500) and HRP-conjugated Goat Anti-Mouse secondary antibody (1:10,000) were used.

In order to confirm the presence of EVs in the isolated fractions, Western blotting was performed to identify the expression of exosomal protein markers. CD63 is a 30–60 kDa lysosomal membrane protein that is composed of four alpha-helical transmembrane domains with two extracellular loops (Jung et al., 2006; Gonzalez et al., 2014). Both the N- and the C-terminus point toward the inside of EVs. In EVs, several tetraspanins, especially CD63, CD81, and CD9, have been used as markers of EVs for the last 2 decades. In our study, endogenous CD63 was monitored as a surface marker for EVs (Kim et al., 2019; Mathieu et al., 2021). Proteins were extracted using the procedure outlined in Section 2.6 from EVs obtained through centrifugation. Our immunoblotting analysis revealed a single band with a molecular weight of approximately 60 kDa (Figure 2 and Supplementary Data S2) in U-937 and THP-1 cells. The band at 60 kDa is most likely due to post-translational modifications (glycosylation of CD63 protein) (Engering et al., 2003). Bands at different positions in the range 30 kDa–85 kDa have been reported which can be due to multiple reasons such as but not limited to post-translational modifications and proteolytic cleavage, and other experimental factors (Engering et al., 2003).

### 3.3 Co-culture of monocytes with EVs during differentiation induction

**FIGURE 3:**
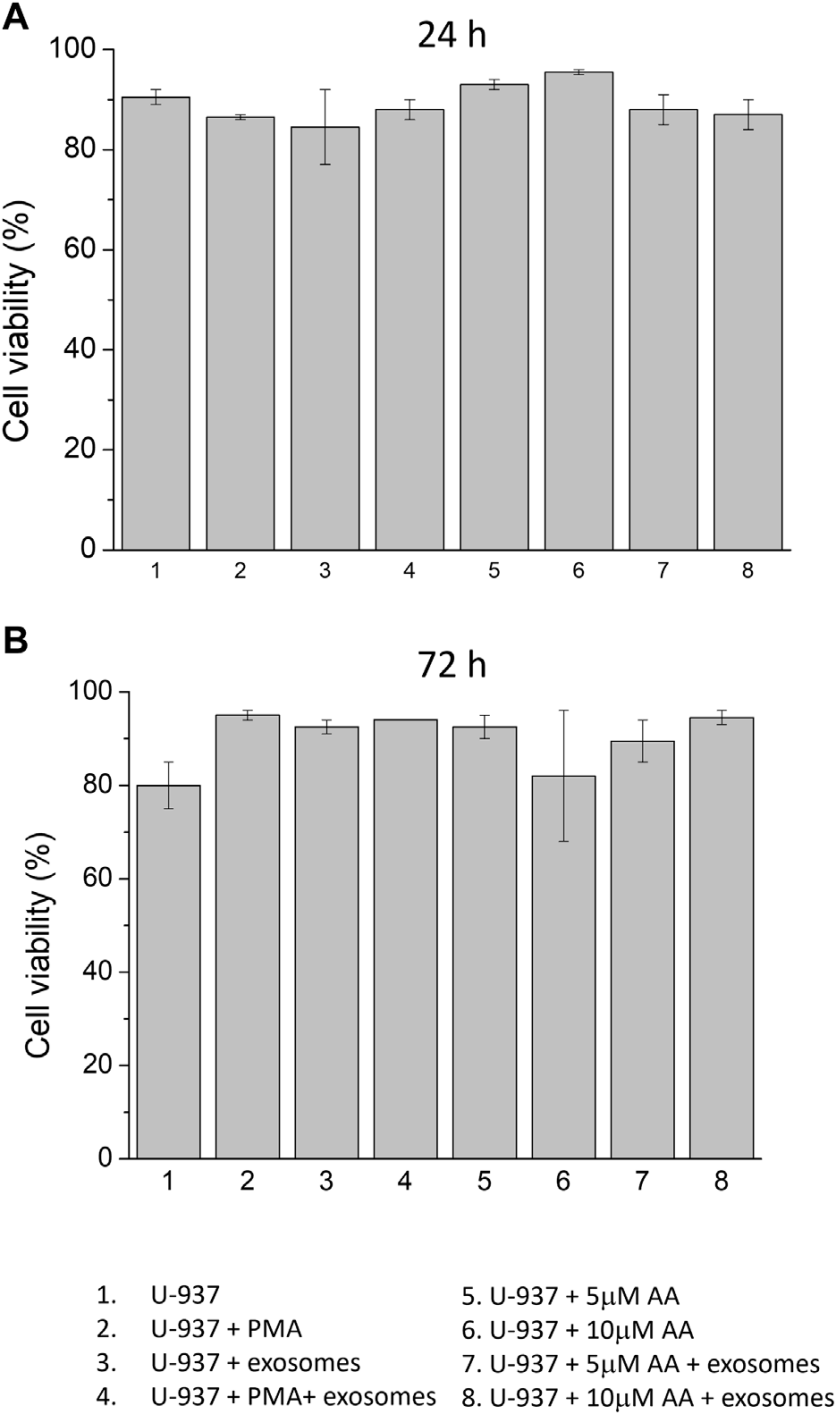
Cell viability of U-937 cells using trypan blue test. U-937 cells were treated with PMA or at different concentrations of ascorbic acid (5 μM and 10 μM) in the absence and presence of exogenous EVs. Cell viabilities were expressed as a percentage at 24 h **(A)** and 72 h **(B)** after treatment. Data are presented as mean value of biological replicates (n = 2).

Cells were treated with differentiation inducers in the absence and presence of EVs (Figure 3). In order to evaluate whether the addition of differentiation inducers led to a change in cell viability of U-937 cells, the trypan blue test was performed in all experimental samples at 24 h and 72 h, including those treated with EVs. In the control (no differentiation agent + no EVs), a viability percentage of 90% and 80% were observed at 24 h and 72 h, respectively. In PMA-differentiated specimens, the recorded viability was 86.5% at 24 h and increased to 95% at 72 h. On the contrary, in samples treated with ascorbic acid, viability remained consistently in the same range, exceeding 80% at various concentrations (5 μM and 10 μM). In EVs treated samples differentiated using PMA, 88% viability was observed at 24 h and 94% at 72 h was recorded. Similarly, in EVs treated samples differentiated using ascorbic acid, it was recorded to be in the same range and maintained above 80% at different concentrations (5 μM and 10 μM); the details have been presented in the form of Table (Supplementary Data S3). On the basis of this dataset, it can be concluded that neither exogenous addition of differentiation agents (either PMA or ascorbic acid) nor supplementation of EVs led to any stress leading to alterations in cell proliferation and viability.

### 3.4 NOX4 and NOX2 expression in monocytes and macrophages

**FIGURE 4:**
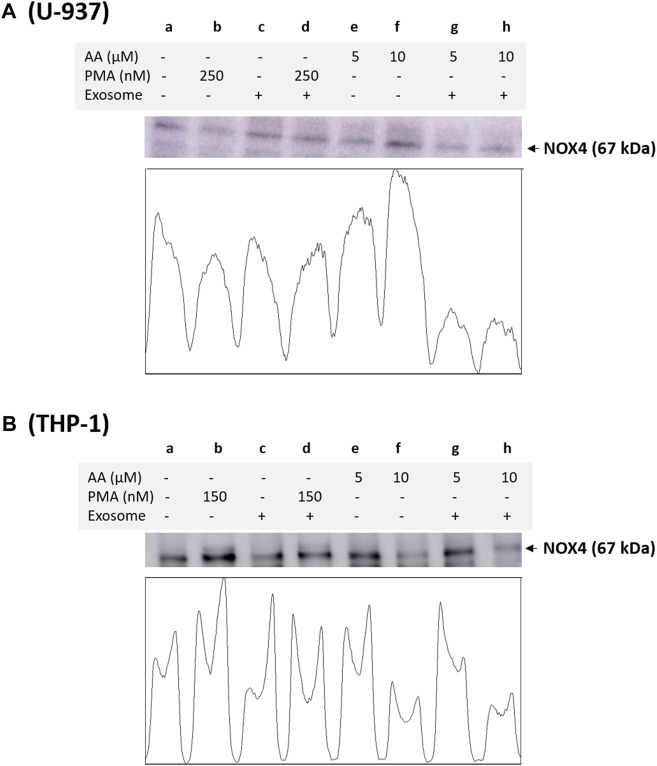
Analysis of NOX4 protein expression in U-937 cells **(A)** and THP-1 cells **(B)**. Control (a) and proteins isolated from EVs co-cultured with cells (c). In b and d, U-937 and THP-1 cells were differentiated using 250 nM PMA and 150 nM PMA, respectively, in the absence and presence of EVs. In lanes e and g, differentiation was induced using 5 μM in the absence and presence of EVs, respectively, while in lanes f and h, 10 μM ascorbic acid was used. The densitometric quantification of NOX4 expression is presented in the lower panel of the figure.

**FIGURE 5:**
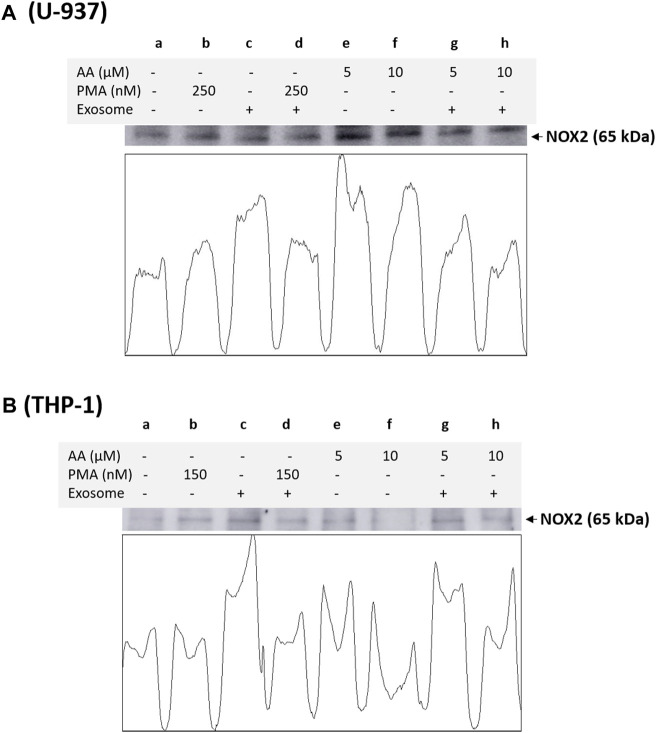
Analysis of catalytic subunit NOX2 of NADPH oxidase in U-937 cells **(A)** and THP-1 cells **(B)**. Control (a) and proteins isolated from EVs co-cultured with U-937 cells (c). In b and d, U-937 and THP-1 cells were differentiated using 250 nM PMA and 150 nM PMA, respectively, in the absence and presence of EVs. In lanes e and f, differentiation was induced using 5 μM in the absence and presence of EVs, respectively, while in lanes f and h, 10 μM ascorbic acid was used. The densitometric quantification of NOX2 expression is presented in the lower panel within the figure.

Based on immunoblotting using NOX4 antibody, we monitored its expression at 67 kDa. In a non-differentiated control, a visible expression of NOX4 can be observed, which is typical for monocytes, as monocytes are well known to express this isoform. In differentiated controls [either PMA or ascorbic acid (5 μM and 10 μM)], the expression of NOX4 is slightly suppressed in all cases except for 10 µM ascorbic acid, which can be hypothesized based on the fact that NOX4 expression is not a characteristic of macrophages. However, under the condition where the EVs are present along with cells, no influence on NOX4 expression is evident compared to the control. Interestingly, in differentiated cells supplemented with EVs for 72 h, a strong suppression of NOX4 expression is evident (especially in ascorbic acid differentiated cells and U-937 cells), which can be hypothesized that when monocytes are transformed to macrophages, NOX4 expression might have been regulated by microRNA (miRNA) (Figure 4). Interestingly, NOX2 expression was found to be only suppressed in macrophages co-cultured with EVs, predominantly in U-937, while in THP-1 cells, no significant suppression was observed (Figure 5). In monocytes co-cultured with EV, an increase in NOX2 expression was observed [Figure 5A (lane c) and Figure 5B (lane c)].

**FIGURE 6:**
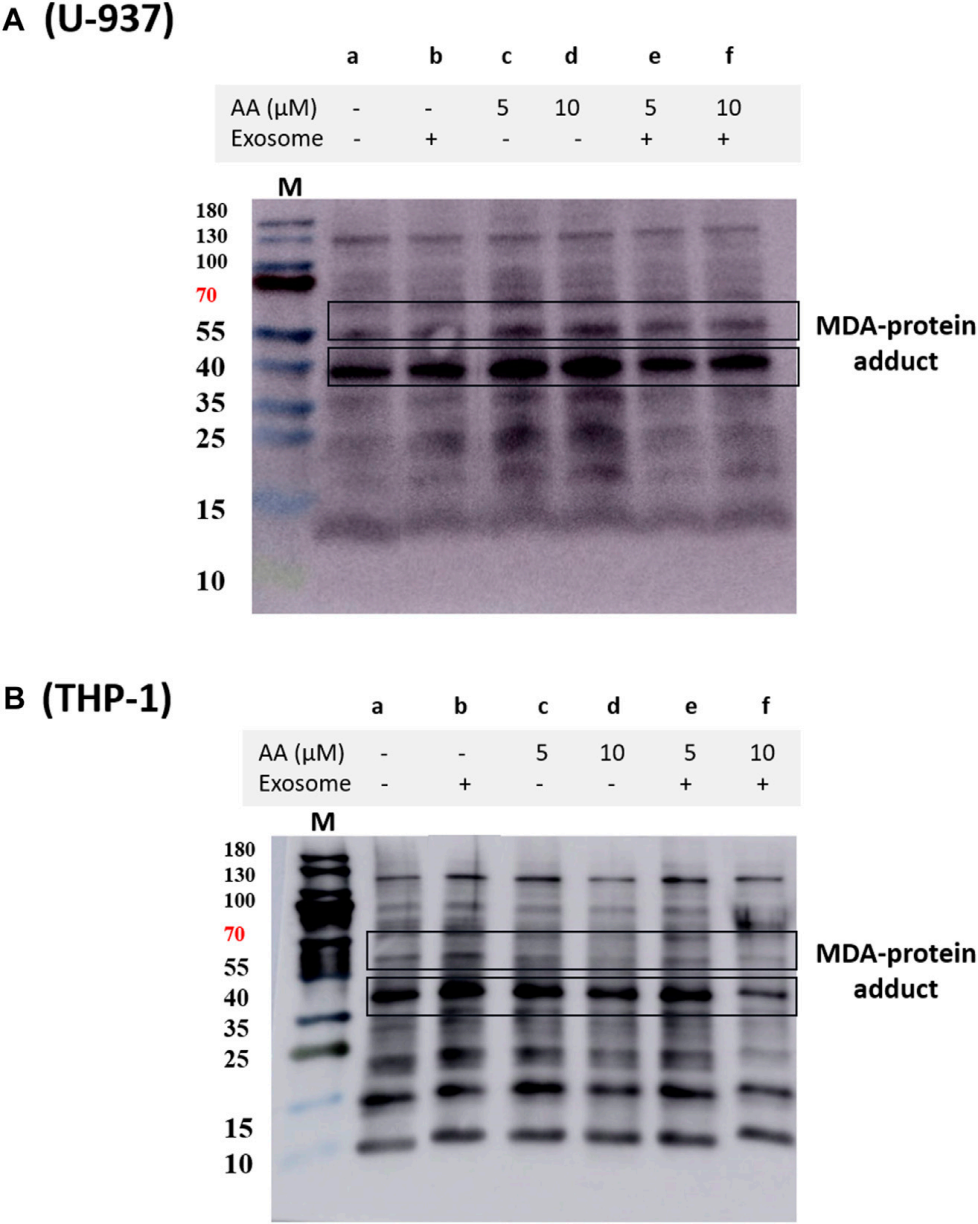
Protein MDA adducts formed in U-937 **(A)** and THP-1 cells **(B)** treated with different inducers. The blot illustrates protein modification in cells that were differentiated for 72 h, visualized using anti-MDA antibody. Lane a shows the control and lane b shows the proteins isolated from EVs co-cultured with cells. In lanes c and e, differentiation was induced using 5 μM in the absence and presence of EVs, respectively, while in d and f, 10 μM ascorbic acid was used.

In THP-1 cells, we validated our findings using immunoprecipitation of NOX2 using NOX2 antibody and protein A agarose beads. NOX2 expression was found to be significantly suppressed in EVs co-cultured macrophages (Supplementary Data S7). We also observed a decrease in overall MDA formation in EVs treated macrophages (Figure 6), which agrees with previous reports (Song et al., 2015; Song et al., 2021).

### 3.5 Crosstalk between EVs and oxidative stress

**FIGURE 7:**
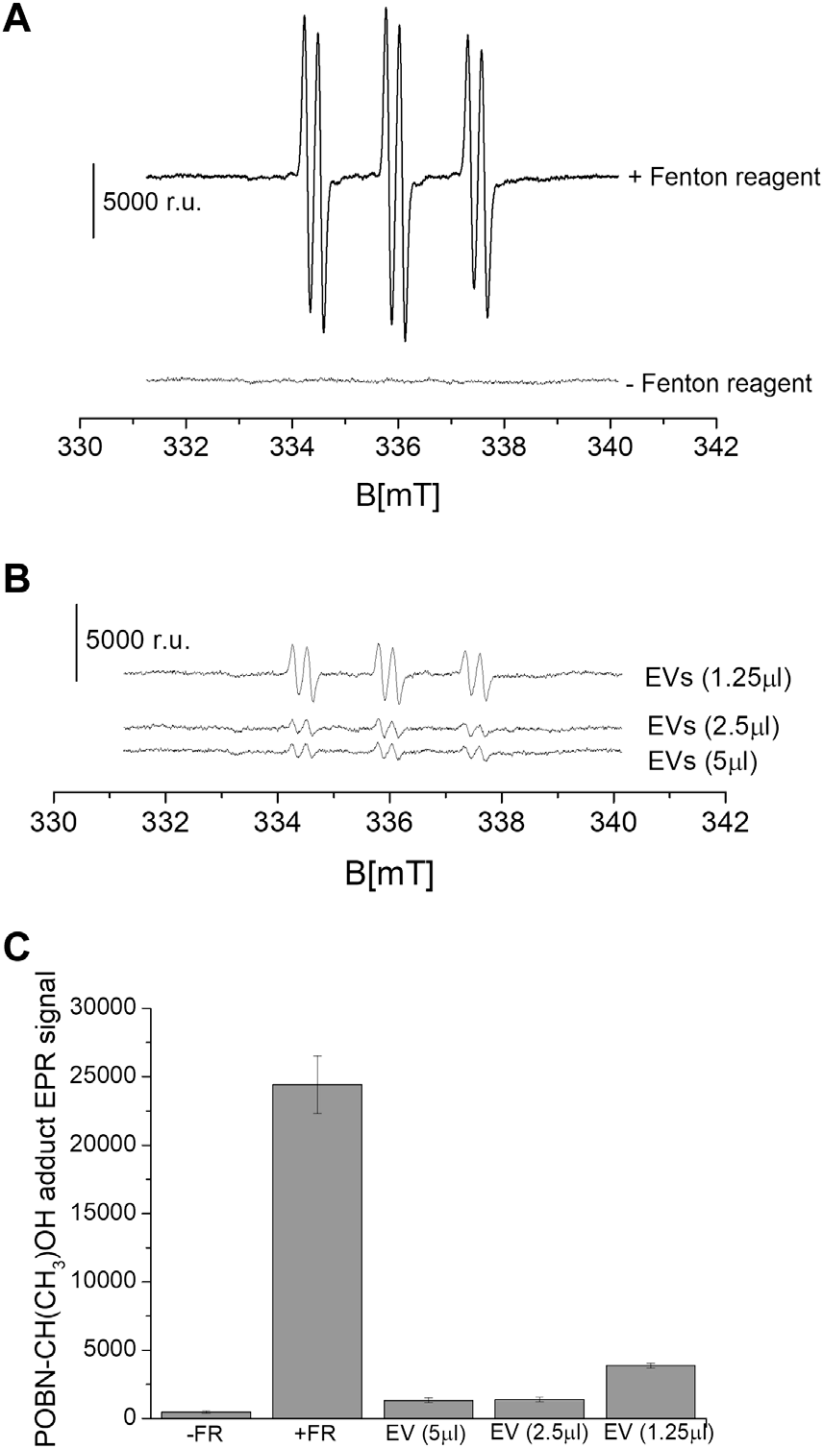
Level of HO^•^ suppression using EPR spin-trapping spectroscopy. Fenton reagent induced POBN (4-pyridyl-1-oxide-N-tert-butylnitrone)-CH(CH_3_)OH adduct EPR spectra were measured in the absence and presence of variable concentrations of EVs isolated from U-937 cell culture. The upper panel **(A)** shows the EPR spectra in controls (absence and presence of Fenton reagent), and the lower panel **(B)** shows the EPR spectra in the presence of Fenton reagent and EVs. In **(C)**, the data are presented as the mean and standard deviation of three measurements.

We measured the suppression of ROS under the exogenous addition of EVs on chemically generated HO^•^. The intensity of the EPR signal was observed in the control samples (Figure 7A). It can be seen that in the sample that did not contain a Fenton reagent (negative control), a relative signal intensity of about ∼800 was observed. Chemically generated HO^•^ using Fenton reagent (0.1 μM FeSO_4_ and 2 mΜ H_2_O_2_) led to a high signal intensity reflecting the formation of α-hydroxyethyl radical adduct of POBN [POBN-CH(CH_3_)OH adduct] (Figure 7A). The intensity of the EPR signal was found to be significantly and linearly suppressed with the exogenous addition of EVs in a dose-dependent manner (Figures 7B, C). The addition of EVs suppressed the signal by up to almost 90%, indicating the antioxidant capacity of the EVs. EVs specifically exosomes mitigate oxidative stress in recipient cells by directly delivering the enzymatic antioxidant [GSH, superoxidedismutase1 (SOD1), thioredoxinreductase1 (TrxR1), methioredoxin reductase (TrxR2) and glutathione peroxidase, among others] or antioxidative enzyme mRNA that later translates aiding to prevention of oxidative stress (Yan et al., 2017; Lin et al., 2022). EVs therapy therefore can be contemplated as an emerging and promising area of regenerative medicine (Muthu et al., 2021; Thakur et al., 2022). EVs through the transfer of cargo to recipient cells can influence cellular functions and therefore, exosomes isolated from different sources can specifically be chosen to target different therapeutic applications. It has been applied in regenerative medicine to promote tissue repairs, in neurological disorders such as Alzheimer’s and Parkinson’s disease, in cancer therapy, among others (Gao et al., 2021; Zhang et al., 2023).

## 4 Conclusion

In our study, monocyte-derived EVs suppressed NOX4 and NOX2 expression in differentiated macrophages, indicating a regulation of NADPH oxidase expression in cells supplemented with EVs from monocytes; the regulation is supposedly at the transcription or translation level. The addition of EVs led to suppression of lipid peroxidation and eventually led to a lower protein modification, which is consistent with recent reports that claim EVs as carriers of antioxidants. Based on the study, EVs can be claimed as a good candidate for therapeutic application in diseases associated with oxidative stress. Typically, the administration of antioxidants in cells is mediated through liposomes preparation. In the case of EVs therapy, this challenge can be easily overcome, as the uptake by cells should be much more efficient.

## Data Availability

The raw data supporting the conclusion of this article will be made available by the authors, without undue reservation.
