# Supplementary material for: NOX2 and NOX4 expression in monocytes and macrophages-extracellular vesicles in signalling and therapeutics
Source: Front Cell Dev Biol. 2024 Apr 16;12:1342227. doi: 10.3389/fcell.2024.1342227 (PMC11058225; doi:10.3389/fcell.2024.1342227)
Supplement: Supplementary file 1 [file Presentation1.pdf]

Supplementary data 1

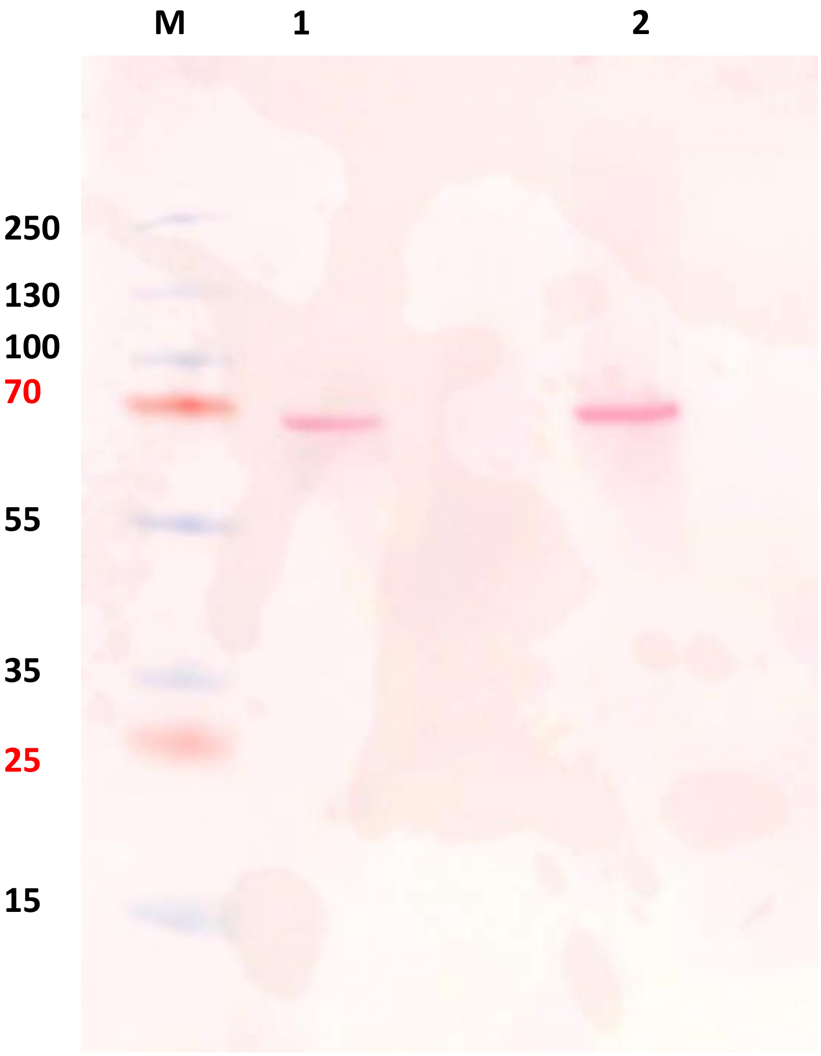

[1] Centrifugation
[2]PEG enrichment

Supplementary data 2 (replicate of Figure 2)

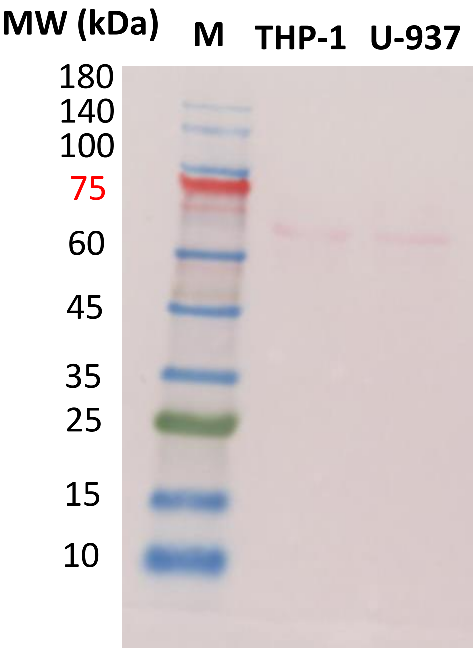

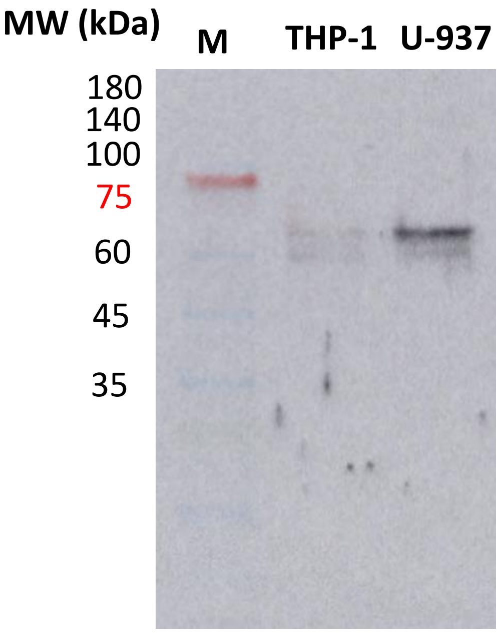

Supplementary data 3

Condition (24h)	total cells/mL	viable cells/mL	viability%		total cells/mL	viable cells/mL	viability%
U937	350000	311000	89		144000	133000	92
U937+PMA	1130000	977000	87		411000	355000	86
U937+Exosome	2140000	1640000	77		422000	389000	92
U937+PMA+Exosome	3400000	2930000	86		511000	461000	90
U937+5μM AA	1270000	1190000	94		417000	383000	92
U937+10μM AA	1730000	1640000	95		1620000	1550000	96
U937+5μM AA+ Exosome	7080000	4450000	91		3770000	3190000	85
U937+10μM AA+ Exosome	7410000	6220000	84		328000	294000	90

Condition (48h)	total cells/mL	viable cells/mL	viability%		total cells/mL	viable cells/mL	viability%
U937	1060000	794000	75		528000	450000	85
U937+PMA	1280000	1190000	96		761000	716000	94
U937+Exosome	816000	744000	91		939000	883000	94
U937+PMA+Exosome	361000	339000	94		372000	350000	94
U937+5μM AA	744000	666000	90		3380000	3210000	95
U937+10μM AA	722000	489000	68		2070000	1990000	96
U937+5μM AA+ Exosome	1790000	1520000	85		1360000	1280000	94
U937+10μM AA+ Exosome	994000	950000	96		1430000	1330000	93

Supplementary data 4- original blot (Figure 4)

A (U-937)

	a	b	c	d	e	f	g	h
AA (µM)	-	-	-	-	5	10	5	10
PMA (nM)	-	250	-	250	-	-	-	-
Exosome	-	-	+	+	-	-	+	+

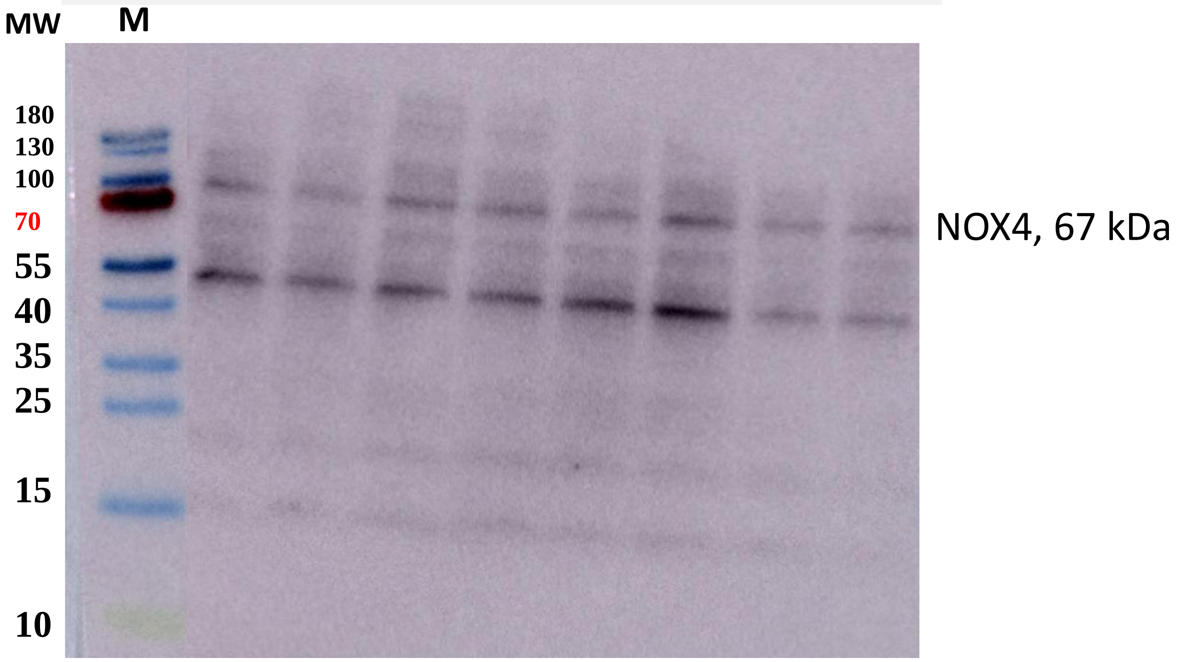

B (THP-1)

	a	b	c	d	e	f	g	h
AA (µM)	-	-	-	-	5	10	5	10
PMA (nM)	-	150	-	150	-	-	-	-
Exosome	-	-	+	+	-	-	+	+

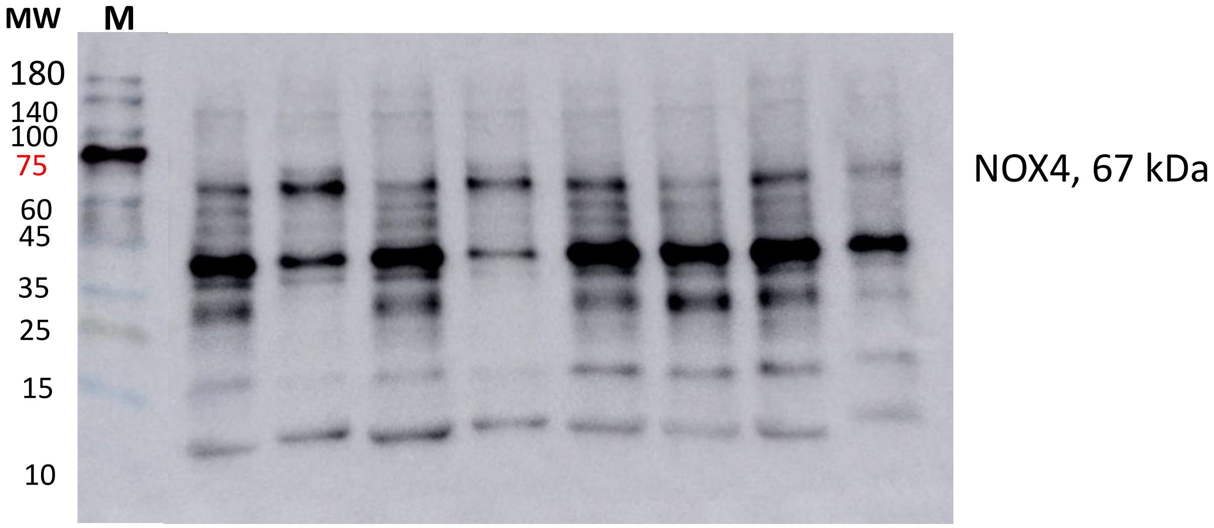

Supplementary data 5- original blot (Figure 5)

A (U-937)

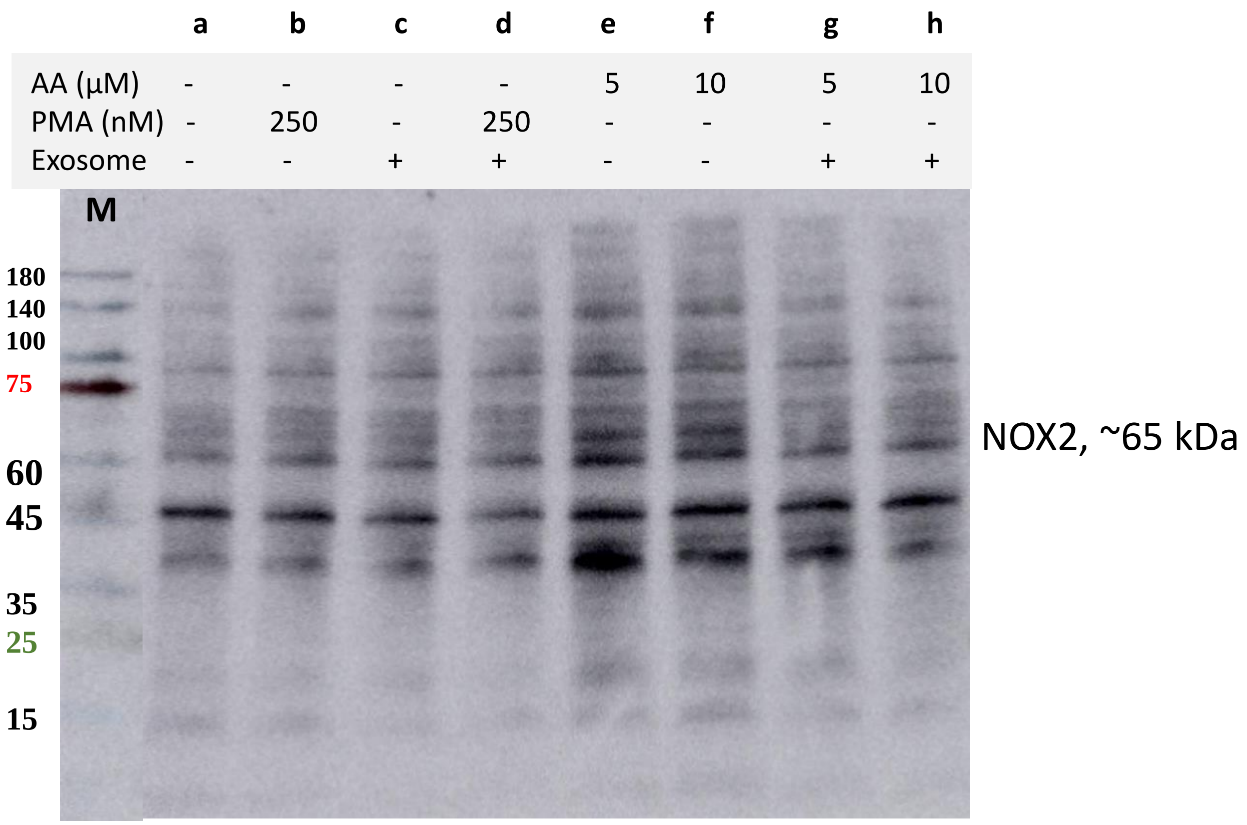

B (THP-1)

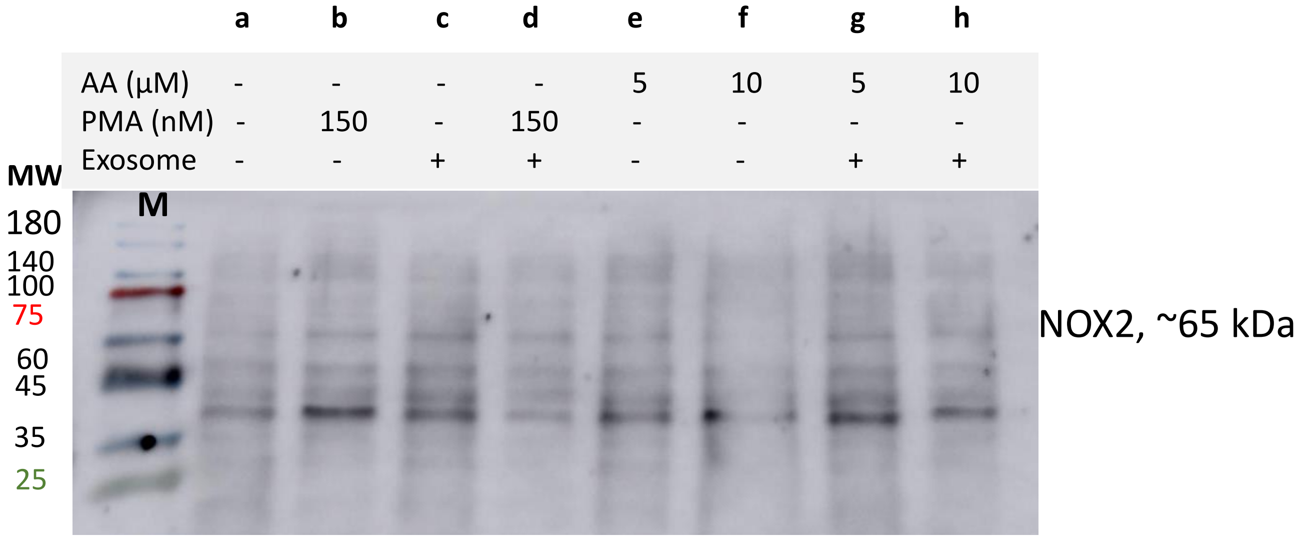

Supplementary data 6- replicate of Figure 5

U-937

	a	b	c	d	e	f	g	h
AA (µM)	-	-	-	-	5	10	5	10
PMA (nM)	-	250	-	250	-	-	-	-
Exosome	-	-	+	+	-	-	+	+

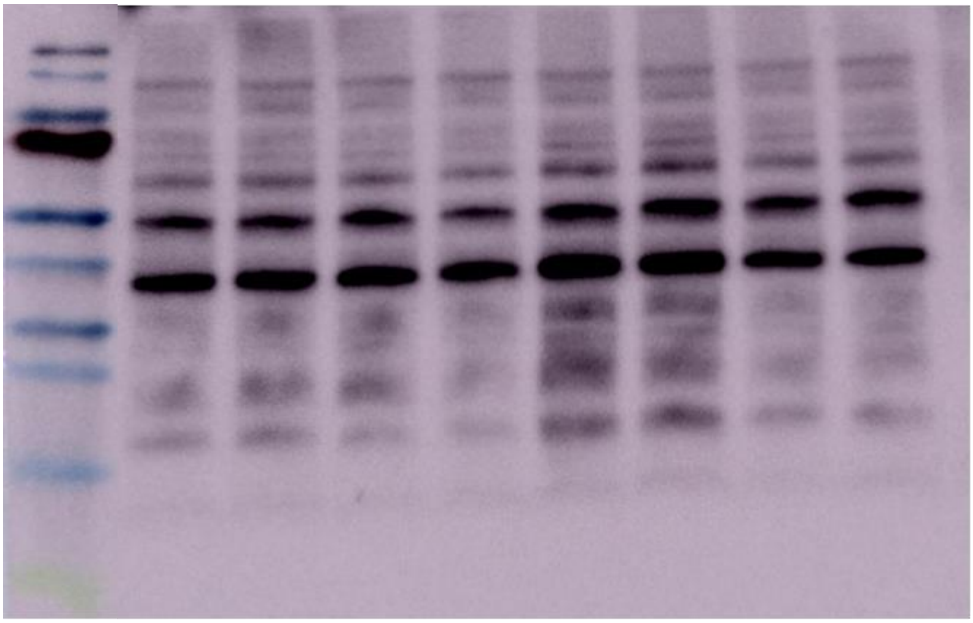

NOX2, ~65 kDa

Supplementary data 7

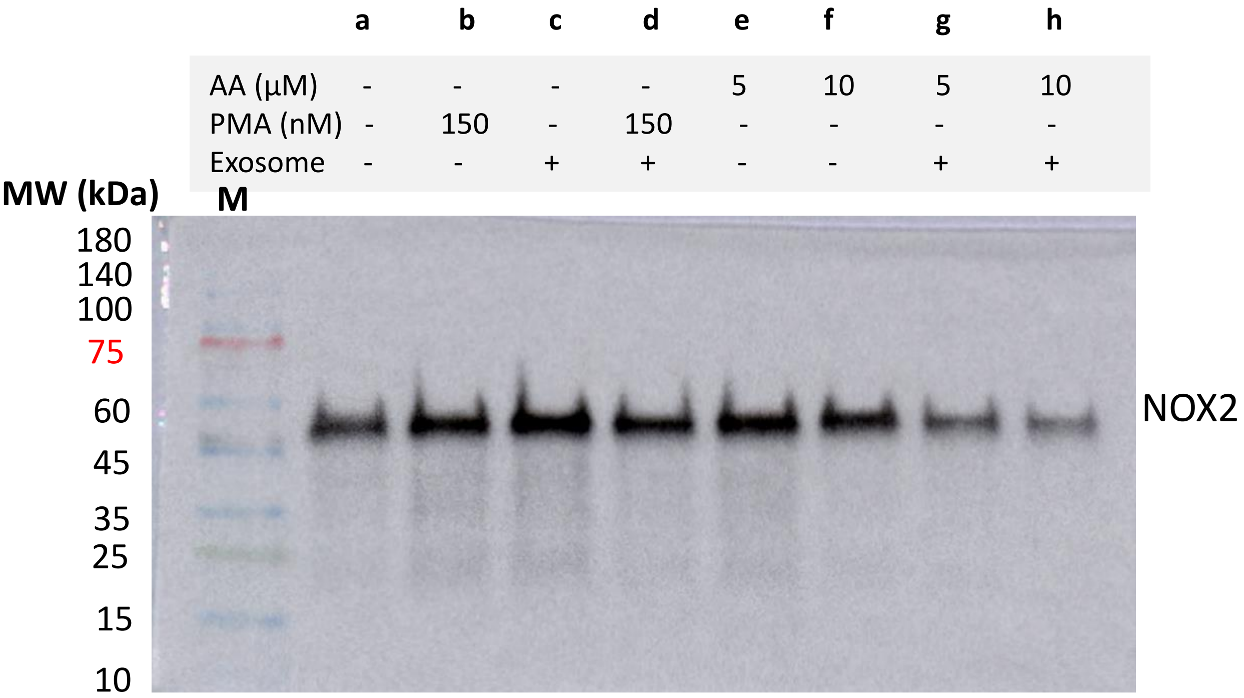

Workflow:

- Protein extraction (as described in manuscript)
- 10 µg protein lysate per variant taken for immunoprecipitation
- Primary antibody (0.25µg, anti-NOX2 antibody)/ sample variant
- Protein lysate and antibody incubated with gentle rocking at 4 ° C overnight.
- Protein A bound to beads (50 µl /mL of cell lysate) added and incubated for 2 hours at 4°C with gentle shaking.
- Washing the beads at 3,000 g for 2 min at 4°C with 1 mL of pre-cooled TBS (collect pellet) (3x).
- Re-suspended in 75µL Tris-buffer saline.
- 25 µL SDS Sample Buffer added; vortex and then centrifuged for 30 s (5000 rpm).
- Sample heated to 100°C for 5 minutes.
- Centrifugation at 10,000 rpm for 2 min.
- Supernatant collected (contains protein of interest) followed by SDS and western blotting.

Supplementary data 8

THP-1

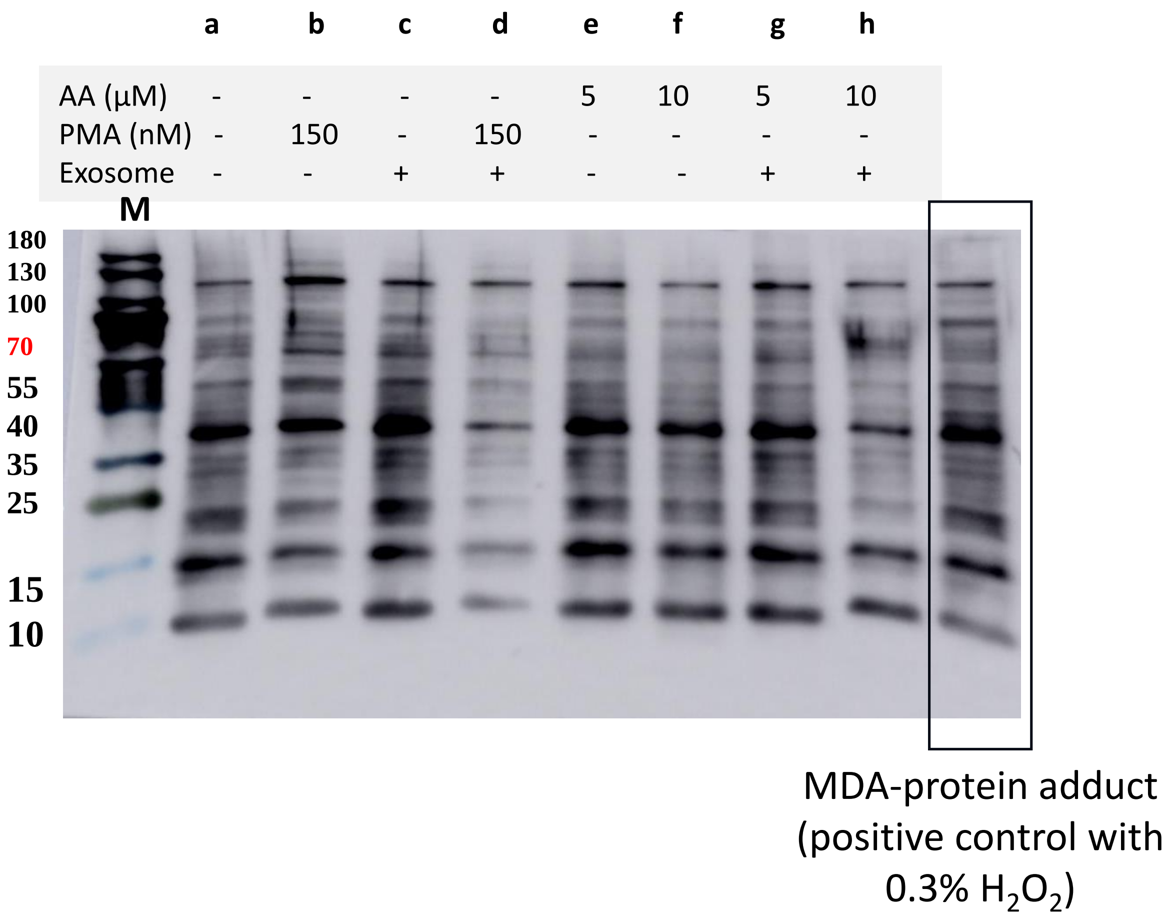

Supplementary data 9

<u>Chemicals and antibodies:</u>

Ponceau S, Sigma Aldrich (P3504)
Trypan blue, Sigma Aldrich (T6146)
RPMI 1640 w/ stable Glutamine - Biosera (LM-R1639)
Fetal Bovine Serum Qualified – Biosera (FB-1090)
Penicillin-Streptomycin-Glutamine Solution 100X - Serana (RAL-001)
Anti-Malondialdehyde polyclonal antibody, Abcam (ab27642)
Goat Anti-Rabbit IgG (H+L)-HRP Conjugate BioRad (1721019)
NOX4- Monoclonal antibody ab 133303 (Abcam)
NOX2 Polyclonal antibody 19013-1-AP (Proteintech)
CD63 Monoclonal antibody (67605-1-Ig) (Proteintech)
CD9 Monoclonal antibody Proteintech (60232-1-Ig)
HRP-conjugated Affinipure Goat Anti-Mouse IgG(H+L) (SA00001-1)
PageRuler Prestained protein ladder- Thermoscientific (26616)
PageRuler Prestained protein ladder- Thermoscientific (26619)
Prestained protein marker - Proteintech (PL00001)
Protein A-Agarose- Santa Cruz Biotechnology (sc-2001)
